# Supplementary figures and images for: Weizmannia Coagulans BC99 Prevents Loperamide-Induced Functional Constipation in Mice Through Increased Intestinal Peristalsis and Modulation of Gut Microbiota Dysbiosis
Source: Nutrients. 2025 May 20;17(10):1729. doi: 10.3390/nu17101729 (PMC12114502; doi:10.3390/nu17101729)

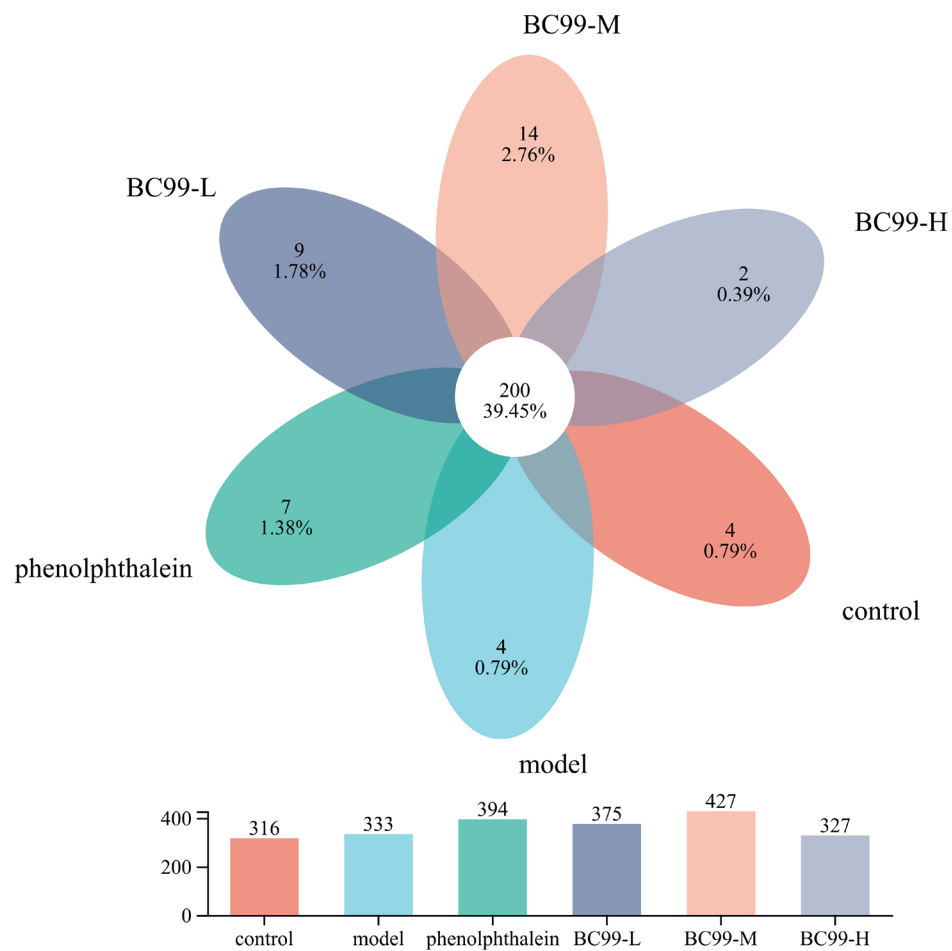

**Figure S1.** Changes in fecal microbiota.

Supplement: Supplementary file 1 [file nutrients-17-01729-s001.zip › nutrients-3584364-supplementary.pdf]
